# Frailty Impact on Periprocedural Outcomes of Atrial Fibrillation Ablation

**DOI:** 10.3390/jcm15010170

**Published:** 2025-12-25

**Authors:** Eran Leshem, Daniel Carny, Adam Folman, Mark Kazatsker, Ariel Roguin, Gilad Margolis

**Affiliations:** Division of Cardiovascular Medicine, Hillel Yaffe Medical Center, Rappaport Faculty of Medicine, Technion—Israel Institute of Technology, Haifa 38100, Israel

**Keywords:** frailty, atrial fibrillation, ablation, outcomes

## Abstract

**Background:** Frail patients undergoing AF ablation face elevated periprocedural risks. However, prior studies often examined composite or long-term outcomes and did not stratify acute complication risks by frailty severity. **Objective:** The objective of this study was to assess the impact of frailty, measured by the Hospital Frailty Risk Score (HFRS) on in-hospital outcomes after AF ablation, and to delineate the risk of specific acute complications across frailty levels. **Methods:** We analyzed a national inpatient cohort of AF ablation hospitalizations (2016–2021). Patients were stratified into low-, intermediate-, and high-frailty groups by HFRS. In-hospital mortality and major complications (stroke, respiratory failure, sepsis, acute dialysis, cardiac arrest, cardiogenic shock) were compared across frailty groups, and multivariable logistic regression identified independent predictors of these outcomes. **Results:** Among an estimated 42,830 AF ablation admissions, 80.0% were low-frailty, 15.0% intermediate, and 5.0% high-frailty. High-frailty patients had markedly higher complication rates than low-frailty patients. In-hospital mortality was 6.1% in high frailty vs. 1.0% in low frailty, and stroke occurred in 4.0% vs. 0.3%, respectively. Rates of respiratory failure (18.0% vs. 3.5%), sepsis (8.0% vs. 1.2%), and acute dialysis (4.0% vs. 0.5%) were also significantly higher in the high-frailty group (all *p* < 0.001). In multivariate analyses, frailty remained a strong independent predictor of complications; high frailty conferred over four-fold higher odds of in-hospital mortality and five-fold higher odds of stroke compared to low frailty. **Conclusions:** Frailty is a powerful predictor of periprocedural complications and mortality in AF ablation patients. Even after accounting for age and comorbidities, patients with higher frailty scores experienced substantially worse in-hospital outcomes. These findings highlight the importance of frailty assessment to identify high-risk patients and inform clinical decision-making for AF ablation.

## 1. Introduction

Catheter ablation is increasingly performed to treat symptomatic atrial fibrillation (AF), offering better rhythm control and improved quality of life compared to medications. However, as the population ages, more frail and medically complex patients are undergoing AF ablation, raising concerns about procedural risks in these individuals. Frailty refers to a syndrome of reduced physiological reserve that makes patients more vulnerable to stressors, and is a recognized predictor of poor outcomes across a wide range of cardiovascular treatments and interventions [1,2]. This vulnerability arises from diminished reserves across multiple organ systems and is particularly prevalent among older adults with CVD, with estimates of frailty as high as 63% in cardiac intensive care unit populations [3,4].

Prior research examining the impact of frailty on AF ablation outcomes using the Hospital Frailty Risk Score (HFRS) [5], focused on an earlier period and primarily reported a composite complication outcome, without providing a granular, multivariable analysis for specific peri-procedural complications. Despite the higher rates of adverse events compared to non-frail patients, the application of catheter ablation for AF to these patients may improve survival and morbidity. Therefore, unanswered questions about the specific risks of in-hospital complications according to frailty severity need to be addressed and offer a more detailed breakdown of outcomes. This broader approach may provide a practical risk profile that could help clinicians in risk stratification prior to proceeding with AF ablation.

Recent studies have examined frailty’s role in longer-term AF outcomes. They found that frail patients had around a six-fold higher risk of AF recurrence after catheter ablation [6]. Moreover, frailty in elderly AF patients was associated with increased all-cause mortality, stroke, and major bleeding [7]. These findings highlight frailty’s prognostic importance beyond the acute hospitalization context.

In the current study, we used the Hospital Frailty Risk Score (HFRS) to stratify AF ablation patients into low-, intermediate-, and high-frailty categories. We hypothesized that higher frailty would be associated with an increased risk of acute complications such as stroke, respiratory failure, sepsis, acute kidney injury, and procedural mortality. Using a large nationwide dataset, we evaluated in-hospital outcomes across frailty levels, providing a detailed complication-specific risk profile that builds on prior knowledge regarding AF ablation in this at-risk population.

## 2. Methods

### 2.1. Data Source

We conducted a retrospective cohort study using the Nationwide Inpatient Sample (NIS) for the years 2016–2021. The NIS is the largest all-payer inpatient database in the U.S., covering a 20% stratified sample of hospital discharges nationwide, and available publicly. Each discharge record includes demographic data, diagnoses, procedures, and outcomes, along with sampling weights to generate national estimates.

### 2.2. Cohort Selection

We identified hospitalizations involving catheter ablation for AF using the appropriate ICD-10 procedure codes (Table S1). In order to avoid inclusion of patients undergoing ablation for other arrhythmias or following CIED implantation (AV node ablation), all other arrhythmias and CIED implants were excluded. Adult patients (age ≥ 18) with a diagnosis of AF who underwent AF ablation during the hospitalization were included. The final unweighted sample comprised 8566 AF ablation hospitalizations over the six-year period. After applying NIS mandatory discharge weights, this corresponded to approximately 42,830 AF ablation admissions nationally.

### 2.3. Frailty Assessment

Frailty was assessed using the Hospital Frailty Risk Score (HFRS) developed by Gilbert et al. [8]. This score is calculated from the presence of 109 ICD-10 diagnosis codes associated with frailty (each contributing 0.1 to 7.1 points) (Table S2). Patients were categorized as low frailty (HFRS < 5), intermediate frailty (5–15), or high frailty (>15) based on established cutoffs, corresponding to non-frail, moderate, and high frailty, respectively. Although other frailty scoring systems like the Norton scale exist, we selected the Hospital Frailty Risk Score (HFRS) due to its prior validation using administrative data and its compatibility with the ICD-10 coding structure of the NIS database [1].

### 2.4. Baseline Variables

We compared baseline patient demographics (age, sex) and comorbidities across frailty groups. Comorbid conditions were identified via ICD-10 codes in the discharge record (Table S1). The Charlson Comorbidity Index (CCI) was calculated for each patient (Table S3) to quantify overall comorbidity burden, although frailty was analyzed separately from the CCI.

### 2.5. Outcomes

The primary outcomes of interest were in-hospital complications occurring during the AF ablation admission. We specifically examined the following major complications: in-hospital mortality; ischemic stroke or transient ischemic attack (new stroke/TIA after the ablation, excluding strokes present on admission); acute respiratory failure (including need for mechanical ventilation); sepsis; acute kidney injury requiring dialysis; cardiac arrest; and cardiogenic shock or use of mechanical circulatory support. These complications were chosen as they represent major adverse events in the context of AF ablation. Minor complications (e.g., minor bleeding or vascular access issues) were not included. Each complication was treated as a binary outcome (present or absent).

### 2.6. Statistical Analysis

Baseline characteristics were compared between frailty groups using one-way analysis of variance (ANOVA) for continuous variables and chi-square tests for categorical variables. A *p*-value < 0.05 was considered statistically significant for these comparisons, with Bonferroni corrections applied for multiple comparisons as appropriate. For outcomes, we first evaluated the unadjusted incidence of each complication in each frailty group and used chi-square tests to assess differences across groups. We then performed a separate multivariable logistic regression model for each complication as the dependent variable. Each model included frailty group (with low frailty as the reference) and the following covariates: age, sex, congestive heart failure (CHF), hypertension, diabetes, and prior stroke/TIA. These covariates were selected due to their potential to confound or mediate the relationship between frailty and complications. Logistic regression results are presented as adjusted odds ratios (aORs) with 95% confidence intervals for intermediate and high frailty (vs. low), as well as for other covariates. Analyses using Stata version 18.0, with a *p*-value < 0.05, were considered significant. The study was exempt from Institutional Review Board approval due to the use of a de-identified national database, and in accordance with the NIS guidelines.

## 3. Results

### 3.1. Patient Characteristics

Among an estimated 42,830 AF ablation hospitalizations in the study period, most patients were classified as low frailty (80.0%), with 15.0% in the intermediate-frailty group and 5.0% in the high-frailty group. Baseline patient characteristics by frailty category are summarized in Table 1. As expected, frail patients were older (mean age 79.4 years in high-frailty group vs. 66.2 years with low frailty; *p* < 0.001) and more often female (58.1% vs. 42.3%; *p* < 0.001) than non-frail patients. They also had a substantially higher prevalence of comorbid conditions such as congestive heart failure (55.3% in high frailty vs. 10.2% in low frailty; *p* < 0.001), prior stroke/TIA (15.2% vs. 4.1%; *p* < 0.001), hypertension (94.1% vs. 76.3%; *p* < 0.001), and diabetes (33.8% vs. 14.0%; *p* < 0.001). Accordingly, the mean Charlson Comorbidity Index increased with frailty (4.1 in high frailty vs. 1.7 in low frailty; *p* < 0.001).

Despite more comorbidities, frail and non-frail patients had similar distributions of AF types, although frail patients were less likely to have AF as the primary reason for admission. Only about 60% of high-frailty patients had AF as a primary diagnosis, compared to 85% of low-frailty patients (*p* < 0.001), suggesting that frail patients were more often admitted for additional conditions and had AF as a secondary issue.

### 3.2. Periprocedural Complications by Frailty

There were notable increases in the incidence of all major complications as frailty increased (Table 2). In-hospital mortality was 1.0% in the low-frailty group, 2.8% in the intermediate group, and 6.1% in the high-frailty group (*p* < 0.001 for trend), representing roughly a six-fold higher unadjusted mortality risk in high- vs. low-frailty patients. Similarly, stroke or TIA occurred in 0.3% of low-frailty patients, 1.1% of intermediate-frailty patients, and 4.0% of high-frailty patients (*p* < 0.001). Acute respiratory failure, one of the most common complications, rose dramatically with increasing frailty: 3.5% in low, 9.8% in intermediate, and 18.0% in high-frailty patients (*p* < 0.001). Sepsis and infectious complications showed a similar pattern, with rates of 1.2%, 4.3%, and 8.0% across low, intermediate, and high-frailty groups, respectively (*p* < 0.001).

Intermediate-frailty patients had complication rates between those of the low- and high-frailty groups, confirming that each step up in frailty category corresponded to a statistically significant increase in complication risk.

**Figure 1:**
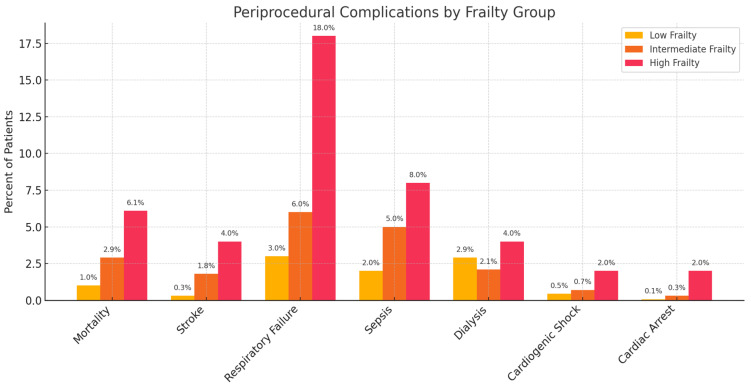
Periprocedural Complications by Frailty Group. Percentage of patients experiencing specific periprocedural complications (including mortality, stroke, respiratory failure, sepsis, dialysis, cardiogenic shock, and cardiac arrest) across three frailty groups: low, intermediate, and high frailty. Frail patients have significantly higher complication rates, particularly in terms of respiratory failure (18.0%) and sepsis (8.0%), compared to those in the low-frailty group. The findings emphasize the importance of frailty as a critical predictor of perioperative risk.

Hemodialysis for acute kidney injury was required in 0.5% of low-frailty patients, 1.9% of intermediate-, and 4.0% of high-frailty patients (*p* < 0.001). Rates of cardiac arrest and cardiogenic shock were likewise higher in frail patients, reaching approximately 3.0% and 2.5%, respectively, in the high-frailty group, versus around 0.5% in the low-frailty group. Overall, the composite incidence of any major complication increased from 7.4% in low-frailty patients to 28.3% in high-frailty patients (*p* < 0.001 for trend). Figure 1 illustrates the proportion of patients experiencing each major complication by frailty group, highlighting the markedly higher complication rates in frail patients, particularly for respiratory failure and sepsis.

### 3.3. Multivariable Analysis

After adjusting for age, sex, congestive heart failure, hypertension, diabetes, and prior stroke, frailty remained an independent and significant predictor of in-hospital complications. As shown in Table 3, patients in the high-frailty group had significantly higher odds of adverse outcomes compared to those with low frailty. High frailty was associated with more than fourfold increased risk of in-hospital mortality and stroke, as well as substantially greater odds of respiratory failure, sepsis, cardiac arrest, and acute kidney injury requiring dialysis. Intermediate frailty also showed increased risks for several complications, though to a lesser extent. Other covariates (female sex, hypertension, diabetes, etc.) did not reach statistical significance in the multivariable models.

**Figure 2:**
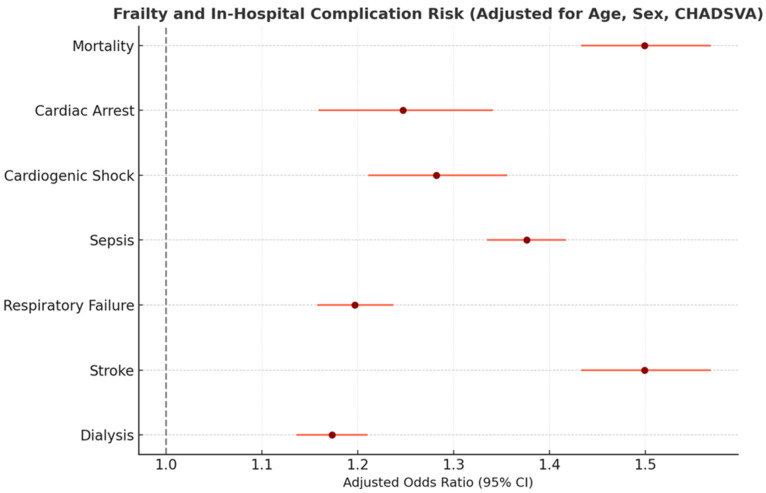
Frailty and In-Hospital Complication Risk. Odds ratios (and 95% CI) for various in-hospital complications as they relate to frailty levels, adjusted for age, sex, and CHADSVAS. Frailty measured by the Hospital Frailty Risk Score (HFRS) increases the risk of complications such as mortality, stroke, respiratory failure, sepsis, and dialysis. Frailty is significantly associated with a higher likelihood of adverse outcomes, even after controlling for other clinical factors. All *p*-values < 0.001.

These findings are visually supported in Figure 2, which presents adjusted odds ratios for key complications across frailty levels. Beyond frailty itself, other predictors emerged: older age was consistently associated with higher risks of mortality, respiratory failure, and sepsis, while congestive heart failure notably increased the odds of respiratory failure and cardiogenic shock. This analysis underscores frailty as a powerful and independent risk factor for adverse outcomes following AF ablation.

## 4. Discussion

In this large nationwide study of AF ablation hospitalizations, we found that frailty, as measured by the Hospital Frailty Risk Score (HFRS), is strongly associated with adverse in-hospital outcomes. Patients with higher frailty scores experienced significantly higher rates of acute procedural complications, including in-hospital mortality, stroke, respiratory failure, sepsis, acute kidney injury, and cardiogenic shock. These findings support the growing recognition that frailty should be incorporated into the preprocedural evaluation of AF ablation candidates to improve risk stratification and inform clinical decision-making.

One important consideration in interpreting these results is the distinction between frailty and traditional comorbidities. While comorbid conditions like heart failure and diabetes are known risk factors for complications, frailty encompasses additional factors such as functional impairment, cognitive decline, and nutritional deficiencies, that are not fully captured by comorbidity indices. Our study demonstrates that frailty remains a significant predictor of complications even after adjusting for age and comorbidities, underscoring its value as an independent risk marker.

This study builds on previous work that explored the link between frailty and outcomes after AF ablation [2]. A prior nationwide analysis using the same dataset and frailty scoring method focused on a shorter time frame and mainly combined all complications into a single outcome [5]. In contrast, we expanded the scope both in terms of duration (covering six years), tested for each major complication separately and adjusted for clinical variables in the multivariable models. Our results provide a clear picture regarding specific elevated risks based on frailty level, information that can be directly useful when discussing treatment risks with patients and tailoring care to individual needs.

Another notable observation is the difference in frailty prevalence between our study and prior research. We found roughly 5% of AF ablation patients to be in the high-frailty category, whereas an analysis of 2017–2019 NIS data reported a frailty rate of only about 0.4%. Several factors may explain this tenfold discrepancy. For one, differences in frailty identification or coding could be at play, as the extraordinarily low fraction in the earlier report suggests a possible data anomaly or a much stricter frailty definition. Additionally, our study included patients with AF as a secondary diagnosis (capturing some frail individuals hospitalized primarily for other conditions and undergoing ablation), while the prior analysis likely focused only on primary AF admissions, inherently selecting a healthier subset of patients. Notably, other contemporary studies have documented frailty in roughly 8% of AF ablation cohorts [2,9,10], which aligns far better with our findings and most probably represents the evolving trend to select sicker patients for an ablation procedure. Therefore, our observed frailty prevalence appears more consistent with real-world expectations, and the markedly lower rate in some previous report most likely reflects methodological differences or an underestimation.

Frailty levels in the overall population, as measured by the HFRS, have shown a clear increasing trend from 2015 to the most recent available data in 2020–2021. Large-scale registry studies demonstrate that the prevalence of frailty, using HFRS, has increased across all older age groups (75, 85, and 95 years) over past decades. The most recent cohorts (2015–2020) showing higher proportions of individuals classified as frail compared to earlier cohorts [11]. Similar trends have been observed in hospital-based studies, where the proportion of patients at intermediate or high frailty risk (by HFRS) has increased over time, although some local fluctuations exist depending on the population and setting [12,13].

Additionally, a single-center analysis also found that frailty was associated with higher complication rates after AF ablation [9]. Most recently, a nationwide study of AF ablation patients confirmed that frailty is a strong predictor of in-hospital adverse events and mortality [10]. However, both of these studies were limited in scope, either by their single-center design or by analyzing frailty as a binary factor without detailed stratification.

Of note, a recent study from the NIS suggested that advanced age and higher frailty are independently associated with adverse outcome [14]. However, despite similarity in results, the methodology differed significantly as the number of AF ablations reported in the same trial period was 7 times the rate observed in our study, and not compatible with prior research, therefore preventing direct comparison.

Our findings should be interpreted in light of external factors during the study years, including the COVID-19 pandemic and evolving ablation technologies. The COVID-19 pandemic coincided with a temporary drop in AF ablation procedures, as many non-urgent cases were deferred. It is plausible that a disproportionate number of high-frailty patients had their ablations postponed during this time. This context is important, as it may have influenced the number and characteristics of frail patients undergoing ablation during those years [15].

Furthermore, the years 2020–2021 saw changes in ablation techniques that could impact complication rates. Clinical practice was shifting, with greater adoption of cryoballoon ablation and the initial introduction of PFA (pulse field ablation). These newer techniques offer different safety profiles including more cardiac selectivity while sparing surrounding structures, and early evidence indicates PFA avoids many of the collateral injuries associated with thermal ablation [16,17]. As ablation technology becomes safer and more efficient, the relationship between frailty and procedural complications may evolve. The presence of these innovations during our study period suggests that continuous re-evaluation of frailty as a risk factor is warranted, as newer ablation strategies might mitigate some of the risks frail patients face.

In clinical practice, assessing frailty in patients being considered for AF ablation may improve patient selection and counseling. Patients with high frailty scores might undergo more intensive prehabilitation, closer monitoring, or consideration of non-invasive management options. Ultimately, tailoring AF ablation decisions and peri-procedural care based on frailty status could potentially reduce complication rates and improve overall patient outcomes. Notably, previous research [18,19] has shown that even very frail patients (for example, those with advanced heart failure) may derive substantial survival benefits from AF ablation. Thus, while frail patients face higher procedural risks, ablation may still be offered in appropriate cases, emphasizing the importance of balancing risks and potential benefits. Additionally, our finding of a significantly higher rate of respiratory failure in frail patients suggests that procedural strategies such as conscious or moderate sedation rather than general anesthesia may potentially help reduce respiratory complications in this population.

## 5. Limitations

As with any observational study, our findings are subject to certain limitations. The use of administrative data means that some clinical details, such as the specific type of ablation procedure or operator experience, were not available. Notably, the NIS does not record procedural success (e.g., restoration or maintenance of sinus rhythm) or long-term post-discharge outcomes, so we could not evaluate ablation efficacy or long-term rhythm outcomes. Also, the NIS does not reliably distinguish AF subtypes (paroxysmal vs. persistent), so we could not examine whether outcomes differ by AF type. Future studies could address frailty–outcome relationships in different AF subpopulations. Additionally, the HFRS is based on ICD-10 codes, which may not fully capture all aspects of frailty. However, the HFRS has been validated in previous studies and has shown good predictive value for adverse outcomes in hospitalized patients.

## 6. Conclusions

Frailty, as quantified by the HFRS, is a powerful predictor of peri-procedural complications in patients undergoing AF ablation. This study provides evidence that frail patients face higher risks of adverse events during hospitalization, even after adjusting for traditional risk factors. Clinicians should consider frailty as part of their pre-procedural risk assessment and explore strategies to optimize frail patients before proceeding with AF ablation. Incorporating frailty screening into clinical practice could help identify high-risk patients and improve overall outcomes in this growing population.

## Figures and Tables

**Table 1:** Baseline Characteristics by Frailty Group (NIS 2016–2021, weighted N ≈ 42,830).

Characteristic	Low Frailty (HFRS < 5) (n = 34,265)	Intermediate Frailty (HFRS 5–15) (n = 6425)	High Frailty (HFRS > 15) (n = 2140)	*p*-Value (Overall)
**Age, mean (SD), years**	66.2 (9.4)	72.8 (8.3)	79.4 (7.8)	<0.001
**Female sex, %**	42.3%	49.8%	58.1%	<0.001
**Congestive heart failure**	10.2%	28.5%	55.3%	<0.001
**Hypertension**	76.3%	88.7%	94.1%	<0.001
**Diabetes mellitus**	14.0%	24.6%	33.8%	<0.001
**Prior stroke/TIA**	4.1%	9.7%	15.2%	<0.001
**Charlson Comorbidity Index, mean**	1.7 (1.1)	3.0 (1.4)	4.1 (1.6)	<0.001
**Primary AF diagnosis (vs secondary)**	85%	72%	60%	<0.001
**Group as % of total cohort**	80.0%	15.0%	5.0%	–

**Table 2:** In-Hospital Complications after AF Ablation, Stratified by Frailty.

Outcome	Low Frailty (HFRS < 5)	Intermediate Frailty (HFRS 5–15)	High Frailty (HFRS > 15)	*p*-Value (Trend)
**In-hospital mortality**	1.0% (≈342/34,265)	2.8% (≈180/6425)	6.1% (≈130/2140)	<0.001
**Ischemic stroke or TIA**	0.3%	1.1%	4.0%	<0.001
**Acute respiratory failure**	3.5%	9.8%	18.0%	<0.001
**Sepsis**	1.2%	4.3%	8.0%	<0.001
**Acute dialysis**	0.5%	1.9%	4.0%	<0.001
**Cardiac arrest**	0.5%	1.1%	3.0%	0.002
**Cardiogenic shock**	0.2%	0.8%	2.5%	0.010
**Any major complication**	7.4%	15.6%	28.3%	<0.001

**Table 3:** Multivariable logistic regression models for each complication outcome (adjusted odds ratios).

Predictor	Intermediate Frailty (vs. Low)	High Frailty (vs. Low)	Female Sex (vs. Male)	Age (per Year)	Heart Failure (CHF)
**Mortality OR (95% CI)**	2.1 (1.3–3.2) **	4.5 (2.8–7.2) **	0.9 (0.7–1.3)	1.05 (1.03–1.07) **	1.8 (1.2–2.7) **
**Stroke OR (95% CI)**	3.2 (1.1–8.5) *	5.5 (2.0–15.2) **	1.5 (0.7–3.1)	1.04 (1.00–1.07) *	1.3 (0.6–2.6)
**Resp. Failure OR (95% CI)**	2.3 (1.9–2.8) **	4.0 (3.1–5.1) **	1.2 (1.0–1.5) *	1.06 (1.05–1.08) **	2.5 (2.1–3.0) **
**Sepsis OR (95% CI)**	2.4 (1.8–3.1) **	3.7 (2.6–5.3) **	0.9 (0.7–1.2)	1.03 (1.01–1.05) **	1.1 (0.8–1.5)
**Dialysis OR (95% CI)**	3.3 (1.8–6.0) **	6.2 (3.2–11.9) **	0.8 (0.4–1.5)	1.04 (1.01–1.07) *	1.4 (0.9–2.3)
**Cardiac Arrest OR (95% CI)**	2.0 (0.9–4.1)	4.1 (1.8–9.3) **	1.1 (0.6–2.0)	1.01 (0.98–1.04)	3.0 (1.7–5.2) **
**Shock OR (95% CI)**	2.8 (0.8–9.5)	5.6 (1.5–20.5) **	0.9 (0.3–2.5)	1.02 (0.98–1.07)	4.5 (2.1–9.5) **

Adjusted odds ratios (ORs) from separate logistic regression models for each outcome. Each model includes frailty category and listed covariates. OR > 1 indicates higher odds of complication. 95% CI = 95% confidence interval. ** *p* < 0.01, * *p* < 0.05. Bold variables are primary variables of interest.

## Data Availability

The data underlying this article will be shared by the corresponding author upon reasonable request, in accordance with institutional policies.

## Supplementary Materials

The following supporting information can be downloaded at: https://www.mdpi.com/article/10.3390/jcm15010170/s1, Table S1: ICD-10-CM/PCS Codes Used for Case Identification and Variables; Table S2: ICD-10 Diagnosis Codes Comprising the Hospital Frailty Risk Score (HFRS) and Their Weights; Table S3: ICD-10-CM Codes for Charlson Comorbidity Conditions and CCI Scores.
