# Supplementary material for: Frailty Impact on Periprocedural Outcomes of Atrial Fibrillation Ablation
Source: J Clin Med. 2025 Dec 25;15(1):170. doi: 10.3390/jcm15010170 (PMC12787278; doi:10.3390/jcm15010170)
Supplement: Supplementary file 1 [file jcm-15-00170-s001.zip › jcm-4037131-supplementary.pdf]

Supplementary

Table S1: ICD-10-CM/PCS Codes Used for Case Identification and Variables

Category	Description	ICD-10 / PCS Codes
**Inclusion Criteria**	Atrial fibrillation diagnosis (primary or secondary)	I480, I481, I482, I4891
	Catheter ablation of AF (procedure codes)	02553ZZ, 02563ZZ, 02573ZZ, 02583ZZ, 02593ZZ, 025F3ZZ, 025G3ZZ, 025H3ZZ, 025J3ZZ, 025K3ZZ, 025L3ZZ, 025M3ZZ, 025S3ZZ, 025T3ZZ
**Exclusion Criteria**		
	I47.0, I47.1, I47.9	Supraventricular tachycardia
	I47.2	Ventricular tachycardia
	I48.3, I48.4, I48.9	Atrial flutter
	I456	Preexcitation
	02HK3JZ, 02HK4JZ, 0JH605Z, 0JH635Z, 0JH606Z, 0JH636Z, 02H64JZ, 02H63JZ, 0JH604Z, 0JH634Z, 0JH804Z, 0JH834Z, 0JH805Z, 0JH835Z, 0JH806Z, 0JH836Z	Pacemaker implantation
	02HK3KZ, 02HK4KZ, 0JH608Z, 0JH638Z, 0JH808Z, 0JH838Z	ICD implantation
	0JH607Z, 0JH637Z, 0JH609Z, 0JH639Z, 02H43JZ, 02H44JZ, 02H40JZ, 02H40NZ,	Cardiac resynchronization therapy implantation

	02H43NZ, 02H44NZ, 0JH807Z, 0JH837Z, 0JH809Z, 0JH839Z	
	Z95.0, Z95.810	History of Pacemaker/ICD
	02580ZZ	Open surgical ablation
**Procedural Complications – Cardiac**	Shock (cardiogenic/unspecified)	T81.11XA, T81.10XA
	Cardiac arrest	I97.12x, I97.7x
	Acute heart failure	I97.11x, I97.13x, I97.19x
	Hemopericardium	I31.2
	Cardiac tamponade	I31.4
**Procedural Complications – Respiratory**	Respiratory failure	J95.82, J95.88, J95.89
	Prolonged mechanical ventilation (>24 hrs)	5A1935Z, 5A1945Z, 5A1955Z
	Reintubation/extended intubation	0B110F4, 0B113F4
**Procedural Complications – Neurologic**	Stroke	I97.81x, I97.82x
**Procedural Complications – Infectious**	Bacteremia / Sepsis	T80.211A, T81.49XA, T81.44XA, T81.12XA, A40.x, A41.x, R65.20, R65.21

Table S2: ICD-10 Diagnosis Codes Comprising the Hospital Frailty Risk Score (HFRS) and Their Weights

This table lists the ICD-10 codes used in calculating the HFRS, along with a brief description and the point weight assigned to each code (per Gilbert *et al.* methodologyfile-jtmsudxh5efgyt7pqfnvvq). Higher weights indicate diagnoses more strongly associated with frailty. A patient's HFRS is the sum of the weights of all frailty-related codes present in their record. (Frailty risk categories: low <5, intermediate 5–15, high >15.)

ICD-10 Code	Description	Weight
F00	Dementia in Alzheimer's disease	7.1
G81	Hemiplegia (paralysis of one side of body)	4.4
G30	Alzheimer's disease	4.0
I69	Sequelae of cerebrovascular disease (late effects of stroke)	3.7
R29	Other symptoms and signs involving the nervous and musculoskeletal systems (e.g., tendency to fall – R29.6)	3.6
N39	Other disorders of urinary system (includes urinary tract infection and incontinence)	3.2

F05	Delirium (not induced by alcohol or other psychoactive substances)	3.2
W19	Unspecified fall	3.2
S00	Superficial injury of head	3.2
R31	Unspecified hematuria (blood in urine)	3.0
B96	Other bacterial agents as cause of disease classified elsewhere (secondary infection cause)	2.9
R41	Other symptoms and signs involving cognitive functions and awareness	2.7
R26	Abnormalities of gait and mobility	2.6
I67	Other cerebrovascular diseases	2.6
R56	Convulsions, not elsewhere classified (e.g., seizures)	2.6
R40	Somnolence, stupor and coma	2.5
T83	Complications of genitourinary prosthetic devices, implants and grafts	2.4
S06	Intracranial injury (head injury with brain involvement)	2.4
S42	Fracture of shoulder and upper arm	2.3
E87	Other disorders of fluid, electrolyte and acid-base balance	2.3
M25	Other joint disorders, not elsewhere classified (e.g., polyarthritis)	2.3
E86	Volume depletion (dehydration)	2.3
R54	Senility (age-related physical debility)	2.2
Z50	Care involving use of rehabilitation procedures	2.1
F03	Unspecified dementia (not otherwise specified)	2.1
W18	Other fall on same level (e.g., slip/trip on surface)	2.1
Z75	Problems related to medical facilities and other health care (e.g., access to care issues)	2.0

F01	Vascular dementia	2.0
S80	Superficial injury of lower leg	2.0
L03	Cellulitis (skin infection)	2.0
H54	Blindness and low vision	1.9
E53	Deficiency of other B-group vitamins	1.9
Z60	Problems related to social environment (e.g., living alone)	1.8
G20	Parkinson's disease	1.8
R55	Syncope and collapse (fainting episodes)	1.8
S22	Fracture of rib(s), sternum and thoracic spine	1.8
K59	Other functional intestinal disorders (e.g., irritable bowel syndrome)	1.8
N17	Acute renal failure (acute kidney injury)	1.8
L89	Decubitus ulcer (pressure ulcer)	1.7
Z22	Carrier of infectious disease (history of infectious disease carriage)	1.7
B95	Streptococcus or Staphylococcus as cause of disease classified elsewhere (infection by these organisms)	1.7
L97	Ulcer of lower limb, not elsewhere classified (e.g., chronic leg ulcer)	1.6
R44	Other symptoms and signs involving general sensations and perception (e.g., hallucinations)	1.6
K26	Duodenal ulcer	1.6
I95	Hypotension (low blood pressure)	1.6
N19	Unspecified renal failure (unspecified chronic kidney failure)	1.6
A41	Other septicemia (sepsis due to unspecified organism)	1.6
Z87	Personal history of other diseases and conditions (past medical history of significant illness)	1.5

J96	Respiratory failure, not elsewhere classified (acute or chronic)	1.5
X59	Exposure to unspecified factor causing injury (accident NOS)	1.5
M19	Other arthrosis (degenerative joint disease of unspecified site)	1.5
G40	Epilepsy (seizure disorder)	1.5
M81	Osteoporosis without pathological fracture	1.4
S72	Fracture of femur (hip fracture)	1.4
S32	Fracture of lumbar spine and pelvis	1.4
E16	Other disorders of pancreatic internal secretion (endocrine disorders of pancreas)	1.4
R94	Abnormal results of function studies (abnormal lab test findings)	1.4
N18	Chronic renal failure (chronic kidney disease)	1.4
R33	Retention of urine (urinary retention)	1.3
R69	Unknown and unspecified causes of morbidity (illness not yet diagnosed)	1.3
N28	Other disorders of kidney and ureter, not elsewhere classified	1.3
R32	Unspecified urinary incontinence	1.2
G31	Other degenerative diseases of nervous system, not elsewhere classified	1.2
Y95	Nosocomial condition (indicator of hospital-acquired condition)	1.2
S09	Other and unspecified injuries of head	1.2
R45	Symptoms and signs involving emotional state (e.g., anxiety, distress)	1.2
G45	Transient cerebral ischemic attacks and related syndromes (TIA)	1.2

Z74	Problems related to care-provider dependency (e.g., need for assistance at home)	1.1
M79	Other soft tissue disorders, not elsewhere classified (e.g., fibromyalgia)	1.1
W06	Fall involving bed	1.1
S01	Open wound of head	1.1
A04	Other bacterial intestinal infections (e.g., foodborne infections)	1.1
A09	Diarrhea and gastroenteritis of presumed infectious origin	1.1
J18	Pneumonia, organism unspecified	1.1
J69	Pneumonitis due to solids and liquids (aspiration pneumonia)	1.0
R47	Speech disturbances, not elsewhere classified (e.g., dysphasia)	1.0
E55	Vitamin D deficiency	1.0
Z93	Artificial opening status (presence of colostomy, ileostomy, etc.)	1.0
R02	Gangrene, not elsewhere classified (e.g., peripheral gangrene)	1.0
R63	Symptoms and signs concerning food and fluid intake (e.g., anorexia)	0.9
H91	Other hearing loss (hearing impairment)	0.9
W10	Fall on and from stairs and steps	0.9
W01	Fall on same level from slipping, tripping and stumbling	0.9
E05	Thyrotoxicosis (hyperthyroidism)	0.9
M41	Scoliosis (spinal curvature)	0.9
R13	Dysphagia (difficulty swallowing)	0.8

Z99	Dependence on enabling machines and devices (e.g., wheelchair, dialysis)	0.8
U80	Resistance to penicillin and related antibiotics (antibiotic resistance)	0.8
M80	Osteoporosis with pathological fracture (fragility fracture)	0.8
K92	Other diseases of digestive system (miscellaneous GI disorders)	0.8
I63	Cerebral infarction (acute ischemic stroke)	0.8
N20	Calculus of kidney and ureter (kidney stones)	0.7
F10	Mental and behavioral disorders due to alcohol use (alcohol abuse)	0.7
Y84	Other medical procedures as cause of abnormal reaction or later complication (iatrogenic)	0.7
R00	Abnormalities of heartbeat (palpitations, arrhythmia symptoms)	0.7
J22	Unspecified acute lower respiratory infection	0.7
Z73	Problems related to life-management difficulty (stress, burnout)	0.6
R79	Other abnormal findings of blood chemistry (abnormal lab results)	0.6
Z91	Personal history of risk factors, not elsewhere classified (e.g., noncompliance with treatment)	0.5
S51	Open wound of forearm	0.5
F32	Depressive episode (major depression)	0.5
M48	Spinal stenosis (secondary code only, indicates spinal stenosis in another context)	0.5
E83	Disorders of mineral metabolism (e.g., electrolyte imbalances)	0.4

M15	Polyarthritis (degenerative)	0.4
D64	Other anemias (e.g., anemia unspecified)	0.4
L08	Other local infections of skin and subcutaneous tissue (e.g., cellulitis not elsewhere classified)	0.4
R11	Nausea and vomiting	0.3
K52	Other noninfective gastroenteritis and colitis (inflammatory diarrhea)	0.3
R50	Fever of unknown origin	0.1

Table S3: ICD-10-CM Codes for Charlson Comorbidity Conditions and CCI Scores

This table lists the ICD-10 codes defining each condition in the Deyo-Charlson Comorbidity Index (CCI), along with the Charlson score assigned to that category. Conditions are counted and weighted according to Charlson's methodology to compute each patient's comorbidity score.

ICD-10 Codes (examples and ranges)	Charlson Comorbidity Condition	CCI Score
I21.x, I22.x, I25.2	Myocardial infarction	1
I11.0, I13.0, I13.2, I25.5, I42.0, I42.5–I42.9, I43.x, I50.x, P29.0	Heart failure	1
I70.x, I71.x, I73.1, I73.8, I73.9, I77.1, I79.0, I79.1, I79.8, K55.1, K55.8, K55.9, Z95.8, Z95.9	Peripheral vascular disease	1
G45.x, G46.x, H34.0x, H34.1x, H34.2x, I60.x–I68.x	Cerebrovascular disease	1
F01.x–F03.x, F04, F05, F06.1, F06.8, G13.2, G13.8, G30.x, G31.0x, G31.1, G31.2, G91.4, G94, R41.81, R54	Dementia	1
J40.x–J47.x, J60.x–J67.x, J68.4, J70.1, J70.3	Chronic pulmonary disease	1
M05.x, M06.x, M31.5, M32.x–M34.x, M35.1, M35.3, M36.0	Rheumatologic disease	1
K25.x–K28.x	Peptic ulcer disease	1
B18.x, K70.0–K70.3, K70.9, K71.3–K71.5, K71.7, K73.x, K74.x, K76.0, K76.2–K76.4, K76.8, K76.9, Z94.4	Mild liver disease	1
E08, E09, E10, E11, E13 + **no** chronic complication codes (E**.0x, .1x, .6x, .8x, .9x)	Diabetes without chronic complications	1

E08, E09, E10, E11, E13 + **with** chronic complication codes (E**.2x, .3x, .4x, .5x)	Diabetes with chronic complications	2
G04.1, G11.4, G80.0, G80.1, G80.2, G81.x, G82.x, G83.x	Hemiplegia or paraplegia	2
C00.x–C14.x, C15.x–C26.x, C30.x–C34.x, C37.x–C41.x, C43.x, C45.x–C58.x, C60.x–C63.x, C76.x, C80.1, C81.x–C85.x, C88.x, C90.x–C96.x	Any malignancy (including leukemia and lymphoma, except non-melanoma skin cancer)	2
I85.0x, I86.4, K70.4x, K71.1x, K72.1x, K72.9x, K76.5, K76.6, K76.7	Moderate or severe liver disease	3
I12.0, I13.11, I13.2, N18.5, N18.6, N19.x, N25.0, Z49.x, Z99.2	Renal disease (end-stage or severe)	3
B20.x	HIV/AIDS (Human immunodeficiency virus infection)	3
C77.x–C79.x, C80.0, C80.2	Metastatic solid tumor	6
Various AIDS-defining illnesses: B37.x, B38.x, B39.x, B45.x, A07.2, A07.3, B25.x, B58.x, B59, A15–A19, A31.x, A81.2, A02.1, B00, C46.x, C81–C96, Z87.01, G93.4x, R64	**Acquired Immunodeficiency Syndrome (AIDS)**	**6**
